# A Novel miRNA Screen Identifies miRNA-4454 as a Candidate Biomarker for Ventricular Fibrosis in Patients with Hypertrophic Cardiomyopathy

**DOI:** 10.3390/biom11111718

**Published:** 2021-11-18

**Authors:** Tilo Thottakara, Natalie Lund, Elisabeth Krämer, Paulus Kirchhof, Lucie Carrier, Monica Patten

**Affiliations:** 1 Department of Cardiology, University Heart and Vascular Center Hamburg, 20253 Hamburg, Germany; t.thottakara@uke.de (T.T.); n.lund@uke.de (N.L.); p.kirchhof@uke.de (P.K.); 2 DZHK (German Centre for Cardiovascular Research), Partner Site Hamburg/Kiel/Lübeck, 20246 Hamburg, Germany; e.kraemer@uke.uni-hamburg.de (E.K.); l.carrier@uke.de (L.C.); 3 Division of Cardiology, Hypertrophic Cardiomyopathy Center of Excellence, University of California, San Francisco, CA 94158, USA; 4 Institute of Experimental Pharmacology and Toxicology, University Medical Center Hamburg-Eppendorf, 20246 Hamburg, Germany; 5 Institute of Cardiovascular Sciences, University of Birmingham, Birmingham B15 2TT, UK

**Keywords:** circulating miRNA, biomarker, ventricular hypertrophy, hypertrophic cardiomyopathy, cardiac fibrosis

## Abstract

(1) Background: Left ventricular hypertrophy, myocardial disarray and interstitial fibrosis are the hallmarks of hypertrophic cardiomyopathy (HCM). Access to the myocardium for diagnostic purposes is limited. Circulating biomolecules reflecting the myocardial disease processes could improve the early detection of HCM. Circulating miRNAs have been found to reflect disease processes in several cardiovascular diseases. (2) Methods: We quantified circulating miRNA molecules in the plasma of 24 HCM and 11 healthy controls using the Human v3 miRNA Expression Assay Kit Code set (Nanostring Tech., Seattle, WA, USA) and validated differentially expressed miRNAs using RT-PCR. (3) Results: In comparison to healthy controls, the levels of six miRNAs (miR-1, miR-3144, miR-4454, miR-495-3p, miR-499a-5p and miR-627-3p) were higher in the plasma of HCM patients than healthy individuals (*p* < 0.05). Of these, higher levels of miR-1, miR-495 and miR-4454 could be validated by real-time PCR. In addition, elevated miR-4454 levels were significantly correlated with cardiac fibrosis, detected by magnetic resonance imaging in HCM patients. (4) Conclusions: Circulating miR-1, miR-495-3p and miR-4454 levels are elevated in the plasma of HCM patients. To the best of our knowledge, this is the first report showing a correlation between miR-4454 levels and cardiac fibrosis in HCM. This suggests miR-4454 as a potential biomarker for fibrosis in these patients.

## 1. Introduction

Hypertrophic Cardiomyopathy (HCM) is a common inherited cardiac condition with a prevalence of 1:200 to 1:500 in the general population [1,2]. It is caused by several mutations in sarcomeric genes leading to myocyte disarray, cardiac remodeling and subsequent ventricular arrhythmia [3]. It is the most frequent cause of sudden cardiac death in young adults and trained athletes, some of which probably remained undiagnosed prior to premature death [4]. HCM phenotypes vary in gene carriers from no or mild disease to severe arrhythmias or heart failure, and approximately one-third of HCM patients do not carry a known genetic defect. Diagnosis of HCM is based on electrocardiogram, history, cardiac imaging using echocardiography, increasingly magnetic resonance imaging (MRI), and genetic testing. Due to the variability of phenotypes, the specialist nature of cardiac imaging, and the fact that the first presentation of HCM can be severe ventricular arrhythmias or even sudden death, blood tests associated with HCM and cardiac fibrosis are desirable to enable early detection and management [4].

A decade ago, several groups succeeded in detecting circulating miRNAs in plasma and serum, raising the question of the potential of miRNAs as biomarkers for various diseases [5,6,7,8,9]. MicroRNAs are small, noncoding single-strand RNA molecules (18–25 nt) acting as posttranscriptional regulators of gene expression. miRNAs regulate the hypertrophic response in the heart [10,11,12]. Furthermore, miRNAs have been studied as biomarkers for myocardial injury and heart failure, for a review [13]. Even though miRNAs as biomarkers for HCM have been studied, often prespecified sets have been evaluated [14].

Therefore, the purpose of this study was to determine potentially relevant miRNA molecules as biomarkers for HCM in a hypothesis-free approach. We analyzed circulating miRNAs using the nCounter Sprint Digital Analyzer (Nanostring Technologies, Seattle, WA, USA) in patients with HCM and controls and related differentially expressed miRNAs to cardiac phenotypes. This technique is based on a direct fluorescent staining of the miRNA molecules and therefore enables the quantification of the authentic levels of all tested circulating miRNAs without the amplification of previously selected target molecules.

## 2. Materials and Methods

### 2.1. Study Population

A total of 24 patients with HCM and 11 healthy controls were recruited in the University Heart Center Hamburg between May 2010 and August 2015. All patients were outpatients at the time of blood sampling. The clinical study protocol was approved by the local ethics board and written informed consent was obtained from all patients and controls. The diagnosis of HCM was based on the current criteria of the European Society of Cardiology guidelines [15]. Plasma samples were collected during an outpatient visit, blood was centrifuged for 10 min with 3000 g at 4 °C. Plasma, buffy-coat and serum were aliquoted and stored at −80 °C. Exclusion criteria were inflammatory, hepatic, respiratory or malignant diseases.

Two-dimensional transthoracic echocardiography was performed using a Philips^®^ iE33 scanner and data were analyzed with Syngo^®^ Dynamics (Siemens Healthcare^®^, Erlangen, Germany). Cardiac magnetic resonance imaging (CMR) was performed using a 1.5T MRI system (Achieva, Philips, The Netherlands). The CMR protocol included cine balanced fast-field sequence imaging acquired in standardized cardiac short-axis and horizontal long-axis planes. After the injection of 0.2 mL/kg gadobenate dimeglumine (Multihance^®^, Bracco Diagnostics, Princeton, NJ, USA), late gadolinium enhancement (LGE) images were acquired using a phase-sensitive inversion recovery sequence in short-axis as well as in two-chamber, three-chamber and four-chamber views. To quantify focal myocardial fibrosis, short axis LGE images were analyzed using cvi42 software (Circle, Calgary, AB, Canada). Endocardial and epicardial contours were drawn. A remote non-LGE region adjacent to the LGE region was chosen and a threshold method with cutoff >5 standard deviations (SDs) above normal myocardium was used. Based on this LGE, mass was calculated and given as gram of fibrosis [16].

### 2.2. miRNA Isolation

Circulating RNA for subsequent direct miRNA measurement using the nCounter Kit was isolated from 500 µL plasma of each participant with the Plasma/Serum Circulating and Exosomal RNA Purification Kit (Slurry Format, #51000, Norgen Biotek, Canada).

To each lysate 1000 attomol of spike-in-miRNA-Oligos [osa-miR414 (UCAUCCUCAUCAUCAUCGUCC)] and cel-miR-254 [(UGCAAAUCUUUCGCGACUGUAGG), Sigma-Aldrich] were added to control for variances in starting material and RNA-extraction efficiency. RNA isolation was performed according to the manufacturer’s protocol. Due to very low RNA yields, all samples were concentrated using RNA Clean & Concentrator-5 Columns (#R1015, Zymo Research, Irvine, CA, USA). RNA Quality Control was performed using the NanoDrop 2000C Spectrophotometer (Thermo Scientific, Waltham, MA, USA). 

For RT-PCR, RNA was isolated using the miRNA Easy Kit (Qiagen, Hilden, Germany) using 200 µL of plasma of each study participant. According to the manufacturer’s instructions, 3.5 µL of diluted syn-spike-in-miR-cel-39 (1.6 × 10^8^/µL Qiagen cat. No. MSY0000010) was added to 200 µL of plasma. The isolation was carried out according to the supplementary protocol for the miRNA Easy Kit (Qiagen Purification of RNA from serum or plasma (RY43 Feb-11). This kit combines phenol/guanidine-based lysis of plasma samples and column-based extraction to purify total RNA. Extracted RNA was eluated in 30 µL of RNase-free water. 

### 2.3. miRNA Screening Using the nCounter Sprint Digital Analyzer

Plasma expression levels of 800 different miRNAs were quantified using the Human v3 miRNA Expression Assay Kit Code set (#CSO-MIR3-12) and nCounter Sprint Digital Analyzer (Nanostring Technologies, Seattle, WA, USA) according to the manufacturer’s conditions. This new sensitive method allows the direct digital detection of miRNA without reverse transcription or amplification. The technology is based on direct molecular barcoding of target molecules through ligation of sequence-specific, color-coded probe pairs to the miRNAs. The probe pair consists of a 3′biotin-labeled capture probe and a 5′fluorescent-labeled reporter probe. Following hybridization and enzymatic purification, the miRNA-probe pair constructs were immobilized on a cartridge for data collection in the nCounter platform [17]. Background subtraction and quality control for 800 miRNAs tested in each sample were performed using the nSolver Software (Nanostring Technologies, Seattle, WA, USA). Low abundant miRNAs with expression levels equivalent to the expression rates of negative controls were excluded from downstream analysis. All samples were normalized using the geometric mean of the two spike-in miRNA Oligos cel-miR-254 and osa-miR-414, and three positive ligation controls were provided in the Code set. 

### 2.4. miRNA Validation by RT-qPCR

To validate the results of the top-6 candidate miRNAs, RT-qPCR was performed with the miRCURY LNA miRNA PCR Kit (Qiagen, Hilden, Germany) on a Quant Studio 6 Flex Real-Time Cycler according to the supplied protocol. Four microliters of eluted RNA were used for RT (miRCURY^®^ LNA^®^ RT Kit). UniSp6 RNA spike-in was added to the master mix as instructed. Three microliters of cDNA (diluted 1:30) templates were used for the subsequent real-time PCR. Appropriate miRCURY LNA miRNA PCR Assays (Qiagen, Hilden, Germany) for the candidate miRNAs were used. CT values of more than 35 were excluded from the analysis. Relative quantification normalized to miR-cel-39 was done using the ΔΔ-Ct method. Results are given in fold change. 

### 2.5. Data Analysis

Statistically significant differences in miRNA levels between two populations (HCM vs. control individuals) were determined using Welch’s T-test. Pearson correlation analysis and linear regression between miRNA levels and clinical parameters were calculated using Prism version 8.2.1. Statistical significance was considered if *p* < 0.05.

## 3. Results

### 3.1. Baseline Characteristics of the Study Population

The analysis of echocardiographic data showed significant thickening of the septal (23 ± 6 vs. 10 ± 1 mm) and inferolateral wall (16 ± 4 vs. 9 ± 1 mm) in HCM patients compared to controls (Table 1). Furthermore, the left ventricular (LV) outflow tract (LVOT) gradient and LV mass were significantly higher in HCM than in controls. Seven of 24 (29%) HCM patients exhibited a rest LVOT gradient >30 mmHg, thus were considered to have HOCM. HCM patients were on different medications as required by the disease state, with some being on multiple medications. Sixty-seven percent were treated with beta blockers, 46% received aspirin/anticoagulation, 21% calcium antagonists, 17% ACE/AT1 inhibitors and 17% diuretics. Amiodaron and clonidine were taken by one patient. Four of the 24 HCM patients (17%) did not take any medication, nor did any of the controls. There were no differences in blood pressure or heart rate between HCM patients and controls (Table 1). Myocardial fibrosis in LGE-CMR was detected in HCM patients but not in the controls. Serum concentrations of high-sensitivity cardiac troponin T (hs-cTnT) and NT-pro-B-type natriuretic peptide (NT-proBNP) were significantly higher in HCM patients than in the controls. Data are summarized in Table 1.

### 3.2. Differentially Expressed of Circulating miRNAs in Patients with HCM

The NanoString nCounter quantified 800 miRNAs. Of these, the levels of six circulating miRNAs (miR-1, miR-3144, miR-4454, miR-495-3p, miR-499a-5p and miR-627-3p) were significantly (*p* < 0.05) higher in plasma from HCM patients than from the controls. No miRNAs with lower plasma concentrations in HCM patients were detected. The miR-3144 exhibited a 2.3-fold higher level in HCM patients than in the control individuals (143 ± 30 vs. 61 ± 5; *p* = 0.01), followed by a 2.1-fold higher level of miR-495-3p (154 ± 30 vs. 74 ± 9) and a 2-fold higher level of miR-627-3p in HCM patients than in the controls (70 ± 14 vs. 34 ± 3; Table 2). Some additional miRNAs with known influence on HCM, such as miR-133a, miR-199a and miR-29a, did not exhibit significantly higher levels in HCM patients than in the controls (Table 2).

### 3.3. Validation of Circulating miRNAs in Patients with HCM

**Figure 1:**
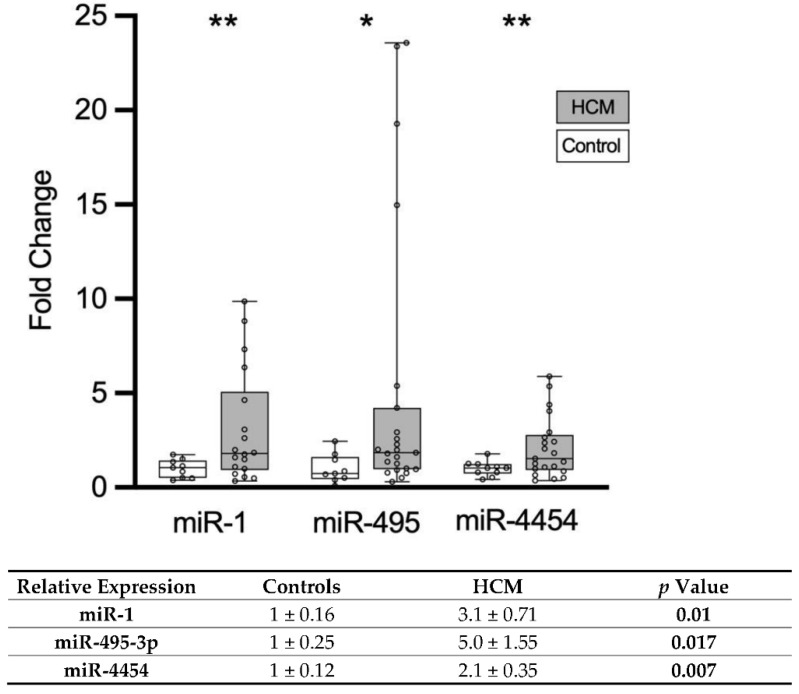
RT-qPCR validation of putative circulating miRNA biomarkers for HCM. Quantification was carried out using the ΔΔ-Ct method. Whisker-plot showing relative expression with median, IQR, minimum and maximum. In the table, the mean of relative expression ± standard error of the mean (SEM) of circulating miRNAs is shown. Three miRNAs could be validated and were upregulated in patients with HCM (white columns) compared to healthy controls (black columns). A *p*-value of <0.05 was considered statistically significant and marked in bold. Data are expressed as mean ± SEM, with * *p* < 0.05 and ** *p* < 0.01 vs. healthy controls, unpaired Welch’s T-test.

All miRNAs showing differential expression on the exploratory screen were quantified by RT-PCR. Of the six putative miRNAs that were identified with the NanoString technology, three were validated by RT-qPCR (Figure 1). The miR-1 level was 3.1-fold higher in HCM patients than in the control individuals (3.10 ± 0.71 vs. 1.00 ± 0.16), miR-495-3p level was 5-fold higher in HCM patients (5.00 ± 1.55 vs. 1.00 ± 0.25 vs., *p* = 0.02) and miR-4454 level was 2.1-fold higher in HCM patients (2.10 ± 0.35 vs. 1.00 ± 0.12). The other three miRNAs could not be detected in the plasma of the patients by RT-qPCR.

### 3.4. Correlation of miRNA Level and Clinical Parameters

**Figure 2:**
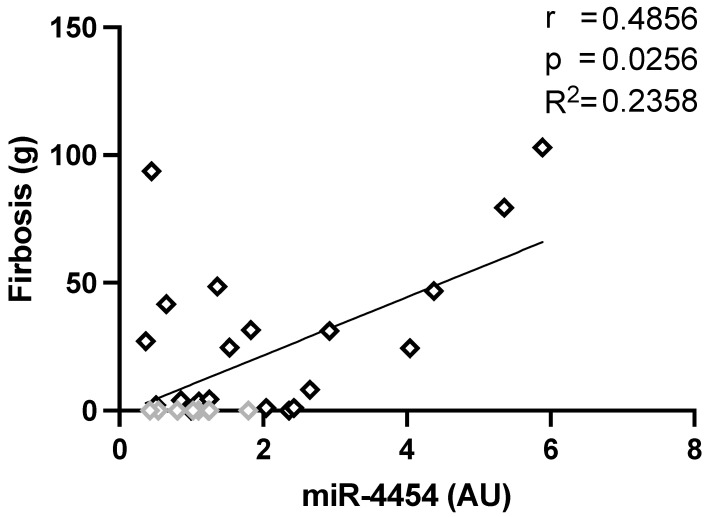
Correlations of LV fibrosis to miRNA-4454 expression in HCM patients. Pearson correlation and linear regression analysis were carried out and a significant correlation of LV fibrosis with the relative expression of miRNA-4454 was determined in HCM patients. For comparison, the controls are given in grey.

The examination of potential direct or inverse correlations of miRNA levels (determined by RT-qPCR) with clinical parameters was carried out. The level of miRNA-4454, but not of miR-1 and miR-495-3p was correlated with the extent of myocardial fibrosis detected by LGE on MRI (r = 0.56, *p* = 0.001) and of septal wall-thickness (r = 0.38, *p* = 0.03, Table 3) in the entire study cohort. There was a significant correlation between miR-1 and miR-495 levels (r =0.59, *p* = 0.001) (Table 3). Based on the correlation shown in the whole collective, we investigated whether there was any correlation in HCM patients. We found a direct correlation between the extent of fibrosis and the relative expression of miR-4454 (r= 0.4856, *p* = 0.0256, R^2^ = 0.2358) (Figure 2) in HCM patients. 

## 4. Discussion

### 4.1. Main Findings of the Study

In this hypothesis-generating study, we could identify and validate three miRNAs being regulated in the plasma of HCM patients using NanoString nCounter as a new screening approach. This is the first study in which miRNA-4454 could be identified as being associated with HCM, and it shows a correlation between miRNA-4454 and hypertrophy and fibrosis in the entire cohort. 

### 4.2. The nCounter Digital Analyzer

This study evaluated the expression pattern of circulating miRNAs in patients with HCM compared to healthy controls using the innovative nCounter platform (Nanostring Technologies, Seattle, WA, USA). This technique allows for the direct digital measurement of isolated miRNAs by a sequence-specific hybridization of every single miRNA molecule with a unique fluorescence code [17]. 

Compared to RT-qPCR, which is considered to be the gold standard of miRNA measurement, the miRNA Expression Assay from Nanostring Technologies does not require reverse-transcription and amplification steps and therefore reflects authentic miRNA molecule counts in the plasma samples. However, sample input per run is limited to a small sample number with the nCounter platform. Therefore, this method is not feasible for high throughput screening. The v3 Human miRNA Expression Assay Code Set Kit provides tags for 800 miRNAs including all published high confidence and clinical miRNAs based on the miRbase, as well as numerous positive and negative controls making it a convenient, easy-to-use, and time-saving miRNA detection method for approaches with small populations, e.g., rare diseases. 

### 4.3. Expression Pattern of Circulating miRNA in Inherited HCM

Several circulating miRNAs are known to be differentially regulated in patients with HCM (e.g., miR-1, miR-133a, miR-199a-5p and miR-29a), some of which, especially miR-29a, are also suggested to be associated with myocardial fibrosis, a hallmark of HCM [18,19,20,21]. All these publications analyzed a predefined “miRNA of interest” using RT-PCR. This method is based on the reverse transcription of isolated RNA into cDNA and subsequent amplification of selective target miRNA molecules using specific primers. Recently, Scolari et al. reviewed the literature on known miRNAs in patients with hypertrophic cardiomyopathy. Eighty-seven miRNAs were described to be differentially expressed in HCM patients, even though there was a large variation in-between all studies. Notably, the authors conclude that there were only a few studies that analyzed phenotype correlations [14].

In our cohort, we found six circulating miRNAs (miR-1, miR-3144, miR-4454, miR-495-3p, miR-499a-5p and miR-627-3p) with significantly higher levels in HCM patients than in the healthy controls. For three of them (miR-1, miR-495-3p and miR-499a-5p), an impact on cardiac diseases was already described [14]. MiR-1 and miR-499 were higher in the circulation of patients with acute myocardial infarction and were suggested to have the potential to be novel, independent biomarkers for cardiac damage [22,23]. Luo et al. found that, in a cardiac-selective miRNA-1-deficient mouse model, a decreased miR-1-expression level resulted in severe LV enlargement and remodeling [24]. The substitution of mir-1 prevented the development of cardiac hypertrophy and remodeling in these miR-1 deficient mice [24]. Since LV hypertrophy is a hallmark of HCM patients, our results suggest that the increased level of miR-1 might also adversely affect cardiac remodeling and function. Therefore, appropriate miR-1 levels seem to play a pivotal role in maintaining myocardial structure and function.

Thus far, two studies show downregulation of miR-1 in cardiac tissue of patients with HCM [25,26]. However, no studies have shown any changes of miR-1 in blood or plasma [14]. One explanation could be chronic myocyte injury in HCM patients leading to higher levels in plasma. Xu et al. described a higher level of circulating miR-499a-3p in patients with atherosclerotic coronary artery disease (CAD). Their results indicated an influence of miR-499a-3p on the proliferation and migration of cardiovascular endothelial and smooth muscle cells by regulating the myocyte enhancer factor 2c (MEF2C) [27]. MEF2 is a prominent transcription factor with a known impact on cardiac development and hypertrophy [28,29]. Different groups evaluated miR-499 levels but none showed differential regulation in HCM patients [21,30]. Interestingly, miR-495 is also MEF2-dependent and was shown to induce proliferation of neonatal mouse cardiomyocytes. Furthermore, the silencing of miRNA-495 attenuated the hypertrophic response of stressed cardiomyocytes in vitro [31]. Since there is a hypertrophic response of cardiomyocytes in HCM, our data suggest that the three miRNAs, miR-1, miR-495 and miR-499a-3p with higher steady-state levels in HCM, may have a direct impact on cardiomyocyte hypertrophy. Further investigations are required to elucidate whether an interaction of miR-499a-3p and/or miR-495 with the MEF2 transcription factor trigger pathophysiological signaling cascades in HCM.

Enhanced levels of three other miRNAs (miR-3144, miR-4454 and miR-627-3p) in HCM patients were detected, none of which were described to have an impact on cardiovascular diseases so far. The miR-627 level was higher in gastric cancer, activated by calcitriol and suppressed CYP3A4 in colon cancer cells and was described to modulate TGF-β1-induced pulmonary fibrosis [32,33,34]. MiR-3144 acts on tumor suppression in high-risk human papilloma virus infection and was recently identified as the regulatory miRNA for RANGRF in human cardiac myocytes, for which, the involvement in cardiac arrhythmia was described [35,36]. MiR-4454 has been reported to be mediating cartilage degeneration and its level in peripheral blood was higher in patients with familial Mediterranean [37,38]. A small study identified miR-4454, amongst many others, as increased after acute myocardial infarction, suggesting a cardiac origin [35]. Furthermore, a cardiac origin was shown in the expression profiling of right and left human atrial appendages, and differential regulation in atrial fibrillation was suggested [39].

In our study, of these six miRNAs only miR-1, miR-495-3p and miR-4454 could be validated with RT-qPCR, probably due to the low expression of the other miRNAs in human plasma as previously proposed [35]. All three showed higher levels in HCM patients than in the controls, with both the nCounter technology and with the RT-qPCR. The first two were previously described in the context of cardiac disease as mentioned above. Although the cardiac involvement of miR-4454 was suggested previously, since its plasma level was increased after myocardial infarction [40], our study is the first to identify miR-4454 as a biomarker for HCM. 

### 4.4. MiR-4454 as a Putative Marker for Cardiac Fibrosis

Since cardiac fibrosis has been associated with cardiac arrythmias, finding a specific marker for cardiac fibrosis is of particular interest [4]. Besides miR-29a, which is commonly accepted as a marker of cardiac fibrosis, no other miRNA has been clearly associated with it [21,41]. MiR-4454 levels correlated directly with the extent of fibrosis and septal LV wall thickness in all study subjects. In contrast, miR-1 or miR-495 levels did not correlate with these. Furthermore, miR-4454 correlated with the extent of fibrosis in HCM patients only, suggesting that it could be a marker of fibrosis in HCM patients. Since myocardial fibrosis is an important predictor of sudden cardiac death in HCM [4], miR-4454 could be used as a novel biomarker for fibrosis and sudden cardiac death in patients with HCM. Further studies need to be carried out to verify this observed relationship in our cohort. Recently, miRNA-4454 was described to be downregulated in ovarian cancer. The authors described a link between the fibroblast microenvironment and the regulation of miR-4454 in ovarian cancer cells. They identified BRG5 and SPARC as target molecules of miR-4454 [42]. According to their data, an increase in miRNA-4454 leads to a reduction in SPARC. SPARC, also known as osteonectin, is a protein that mediates cell–matrix interactions, regulating extracellular matrix and collagen homeostasis. Several studies evaluated SPARC in the heart and showed that SPARC-null or SPARC-deficient animals had blunted age-related changes to collagen and cardiac stiffness, whereas overexpression leads to a cardiomyopathy phenotype [43,44]. Further studies should evaluate the interaction of miR-4454 and SPARC in the context of HCM.

### 4.5. Limitations of the Study

This study has limitations. First, the number of HCM patients was small. Second, since circulating miRNAs were analyzed in patient plasma, the miRNA origin and their potential role in cardiac diseases remain to be determined in future studies. Therefore, our results should be considered as a hypothesis-generating study that needs to be validated in larger cohorts. All presented miRNA levels were measured in plasma samples. The origins of these circulating miRNA molecules are unknown and can only be conjectured based on tissue-specific expression (e.g., muscle-specificity of miR-1).

## 5. Conclusions

With the innovative nCounter digital analyzer, we identified six miRNAs that exhibit higher levels in the plasma of HCM patients than in controls. Three of them were validated by RT-qPCR. In our cohort, miRNA-4454 directly correlated with the septal LV wall thickness and the extent of fibrosis. Further, miRNA-4454 correlates with the extent of fibrosis in HCM patients. These results suggest that miR-4454 might serve as a potential biomarker for myocardial fibrosis in hypertrophic cardiomyopathy.

## Figures and Tables

**Table 1:** Baseline characteristics of patients with HCM and healthy controls.

	HCM	Controls	*p* Value
Total (n)	24	11	
Age (y)	54 ± 14	49 ± 9	0.16
Sex (female)	12 (50%)	6 (55%)	>0.99
Height (cm)	170 ± 10	173 ± 12	0.45
Weight (kg)	80 ± 14	75 ± 15	0.43
Septum thickness (mm)	23 ± 6	10 ± 1	**<0.001**
Inferolateral wall thickness (mm)	16 ± 4	9 ± 1	**<0.001**
Max. LVOT gradient (mmHg)	29 ± 29 (19; 29; 29%)	5 ± 2	**<0.001**
Fibrosis (g)	26 ± 30	0	**<0.001**
Hs-cTnT (pg/mL)	32 ± 63	4 ± 3	**0.04**
NT-proBNP (ng/mL)	1646 ± 2150	49 ± 33	**0.002**
AF (n)	4 (17%)	0 (0%)	0.28
VT/Syncope (n)	14 (58%)	0 (0%)	**<0.001**
Blood pressure (mmHg)	126/74	127/79	0.87/0.21
Heart Rate (bpm)	69 ± 13	68 ± 9	0.80

Data are given as N (%) for binary variables and as mean ± standard deviation for continuous variables. Abbreviations: AF, atrial fibrillation; BMI, body mass index; hs-cTnT, high-sensitivity cardiac troponin T; LV, left ventricular; LVOT, left ventricular outflow tract gradient; NT-proBNP, pro-B-type natriuretic peptide; VT, ventricular tachycardia. For max LVTOT gradient median, IQR, the proportion of patients with LVOT gradient >30 mmHg is given. A *p*-value of <0.05 was considered statistically significant and marked in bold. Data are expressed as mean ± SD, unpaired Welch’s T-test for continuous data and Fisher’s exact test for categorical data.

**Table 2:** Screening of circulating miRNAs in hypertrophic Cardiomyopathy (nCounter). The absolute fluorescence counts of circulating miRNAs are shown. Of the 800 miRNAs quantified, six miRNAs exhibited higher plasma levels in patients with hypertrophic cardiomyopathy than in the healthy control individuals.

miRNA	Controls	HCM	*p* Value
miR-1	31 ± 3	58 ± 10	**0.02**
miR-3144	61 ± 5	143 ± 30	**0.01**
miR-4454	62 ± 8	123 ± 25	**0.03**
miR-495-3p	74 ± 9	154 ± 30	**0.02**
miR-499a-5p	75 ± 9	133 ± 24	**0.04**
miR-627-3p	34 ± 3	70 ± 14	**0.02**
miR-133a	28 ± 5	51 ± 13	0.11
miR-199a-5p	21 ± 2	36 ± 7	0.06
miR-29a	21 ± 3	32 ± 6	0.09

Data are expressed as mean ± SEM, with *p* < 0.05 (bold) vs. controls, unpaired Student’s T-test.

**Table 3:** Direct correlation of miRNA-4454 with fibrosis and septal wall thickness in entire cohort.

	miR-1	miR-495-3p	miR-4454
Fibrosis (LGE-CMR) g	r = −0.21 *p* = 0.29	r = −0.12 *p* = 0.51	**r = 0.56** ***p*** ** = 0.001**
LV-septal wall thickness mm	r = 0.2 *p* = 0.32	r = −0.07 *p* = 0.71	**r = 0.38** ***p*** ** = 0.03**
LV mass g	r = 0.21 *p* = 0.33	r = 0.08 *p* = 0.7	r = 0.34 *p* = 0.09
LVOT Gradient mmHg	r = −0.1 *p* = 0.61	r = −0.03 *p* = 0.88	r = 0.32 *p* = 0.08
hsTNT pg/mL	r = −0.11 *p* = 0.61	r = −0.07 *p* = 0.71	r = 0.01 *p* = 0.97
NT-proBNP ng/L	r = −0.13 *p* = 0.51	r = −0.14 *p* = 0.44	r = 0.07 *p* = 0.69
Age years	r = 0.03 *p* = 0.87	r = 0.03 *p* = 0.88	r = 0.06 *p* = 0.77
Sex m/f	r = −0.28 *p* = 0.16	r = −0.07 *p* = 0.7	r = 0.19 *p* = 0.29
Height m	r = 0.28 *p* = 0.16	r = −0.01 *p* = 0.95	r = −0.15 *p* = 0.43
Weight kg	r = 0.26 *p* = 0.19	r = −0.13 *p* = 0.49	r = 0.32 *p* = 0.09
BSA (m/100)^2^	r = 0.28 *p* = 0.16	r = −0.01 *p* = 0.96	r = −0.16 *p* = 0.4
AF yes/no	r = 0.03 *p* = 0.88	r = 0.07 *p* = 0.7	r = −0.25 *p* = 0.18
VT/Synkope yes/no	r = −0.02 *p* = 0.94	r = −0.1 *p* = 0.58	r = 0.28 *p* =0.12
miR-495-3p	**r = 0.59** ***p*** ** = 0.001**	–	r = 0.11 *p* = 0.58
miR-1	**–**	**r = 0.59** ***p*** ** = 0.001**	r = 0.06 *p* = 0.79

Pearson correlation analysis shows a significant direct correlation of the miRNA-4454 with fibrosis and septal wall thickness in our entire cohort (marked in bold). miR-1 and miR-495-3p correlate with each other. *p*-values < 0.05 were considered as statistically significant. AF: atrial fibrillation, LGE-CMR: late gadolinium enhancement cardiac magnetic imaging, LV: left ventricle, LVOT: left ventricular outflow tract, VT: ventricular tachycardia.

## Data Availability

The data presented in this study is available from GEO (Accession-Number GSE188324).
